# *Listeria* Species Occurrence and Associated Risk Factors and Antibiogram of *Listeria Monocytogenes* in Milk and Milk Products in Ambo, Holeta, and Bako Towns, Oromia Regional State, Ethiopia

**DOI:** 10.1155/2022/5643478

**Published:** 2022-04-14

**Authors:** Bizunesh Mideksa Borena, Lemma Dilgasa, Endrias Zewdu Gebremedhin, Edilu Jorga Sarba, Lencho Megersa Marami, Kebede Abdisa Kelbesa, Nega Desalegn Tadese

**Affiliations:** ^1^Department of Veterinary Sciences, School of Veterinary Medicine, Ambo University, P.O. Box 19, Ambo, Ethiopia; ^2^Department of Veterinary Laboratory Technology, School of Veterinary Medicine, Ambo University, P.O. Box 19, Ambo, Ethiopia

## Abstract

A cross-sectional study was conducted to estimate the prevalence and associated risk factors of *Listeria* species and assess the antibiogram of *Listeria monocytogenes* (*L. monocytogenes*) isolated from milk and milk products from Holeta, Ambo, and Bako towns, Ethiopia. A total of 482 samples (384 milk, 35 cottage cheeses, 30 bulk tank milk, and 33 curdle milk) were collected using a systematic random sampling method and isolation and identification of *Listeria* species were done using standard microbiological techniques. An antimicrobial susceptibility test for *L. monocytogenes* was performed using the Kirby–Bauer disk diffusion technique. Descriptive statistics were used to summarize the prevalence of *Listeria,* while the Chi-square test and logistic regression were used to determine the association between the prevalence of *Listeria* and the risk factors and the magnitude of association, respectively. The overall isolation rate of *Listeria* species from milk and milk products was 7.67% (37/482; 95% confidence interval (CI): 5.46, 10.42). The highest prevalence of *Listeria* species (15.15%; 95% CI: 5.11–31.90) was detected in bulk tank milk and the lowest prevalence of *Listeria* species (6.67%; 95% CI: 0.82–22.07) and *L. monocytogenes* (0.00; 95% CI: 0.00–1.15) was found in curdled milk. The other species isolated were *Listeria welshimeri* 0.62% (3/482; 95% CI: 0.13–1.81), *Listeria seeligeri* 1.04% (5/482; 95% CI: 0.33–2.40), *Listeria ivanovi* 1.24%, (6/482; 95% CI: 0.45–2.68), and *Listeria grayi* 2.49% (12/482; 95% CI: 5.46–10.42). Univariable logistic regression showed that study town, herd size, farm size, number of lactating cows, and management system were the factors significantly associated with the isolation of *Listeria* species at farm level, while the intensive management system was the independent predictor at cow level in the multivariable model (adjusted odds ratio = 3.38, *P*=0.046). *L. monocytogenes* isolates showed the highest resistance against oxacillin (100%), amoxicillin (90.91%), and vancomycine (81.82%). *L. monocytogenes* showed a very high multidrug resistance (MDR) [81.82%]. In conclusion, the current study showed the widespread type of *Listeria* species MDR *L. monocytogenes* isolates in cow raw milk and milk products from Ambo, Holeta, and Bako towns, Oromia Regional State, Ethiopia.

## 1. Introduction

Biological contamination of food is a global public health problem. Among animal-origin foods, cow's milk needs special attention because it is highly perishable and widely consumed by the population. There is a trend to consume raw milk based on the idea that heat destroys the nutritional and health benefits of milk and can cause detrimental effects [1]. On a worldwide basis, over 91 million people are sickened as a consequence of food-borne pathogens and associated illnesses. Food-borne pathogens and outbreaks of associated diseases occur frequently and pose significant constraints to consumer health in many parts of the world, resulting in morbidity, mortality, and economic losses [2]. Through food, more than 200 food-borne diseases are transmitted to humans. Listeriosis is one of the severe food-borne diseases caused by consuming food contaminated with *Listeria monocytogenes* [3]. It is associated with the consumption of contaminated milk, soft or semisoft cheese, undercooked and ready-to-eat foods, and unwashed raw vegetables and fruits [4].

The safety of dairy products concerning food-borne diseases in developing countries is of great issue. This is due to the poor practices and the production of milk and milk products under unhygienic conditions [5]. Raw milk may be a risk for public health if contaminated with zoonotic pathogens, which are often part of the intestinal ﬂora or present on the udder of healthy dairy animals and can easily contaminate the environment and the milk during the production process [6]. Three factors play a fundamental role in food poisoning outbreaks concerning food handlers: knowledge, attitude, and practice [7].

Previous studies conducted in different parts of Ethiopia showed different prevalence values and drug resistance profiles of *Listeria* from milk and milk products. Accordingly, *Listeria* species were reported from raw milk in Selale (22%) by Gebretsadik et al. [8] and in Jimma town by Muhammed et al. [9]. In addition, Derra et al. [10] showed the prevalence of *Listeria* from raw milk 8.5%, cottage cheese 6.8%, and cream cake samples 22.0%, and a low level of occurrence of antimicrobial-resistant *L. monocytogenes* isolates from Addis Ababa. *L. monocytogenes* has also been reported in several milk processing environments at various stages of production [11], leading to higher chances of cross-contamination of the ﬁnished products [12]. Garedew et al. [13] from Gondar town reported *Listeria* species prevalence of 25% in food samples including unpasteurized milk. The same authors also detected multidrug resistant *L. monocytogenes*. Another study conducted in the central highlands of Ethiopia showed a 28.4% overall prevalence of *Listeria* spp. and 5.6% *L. monocytogenes* in raw milk and milk products [14].

Reinforcement of pathogenic Listeria species load reduction regulation, food safety education, food trade and food handler awareness, increasing awareness concerning health impacts of listeriosis, regular disease follow-up, and early investigation of reported diseases are some of the effective strategies to control listeriosis. [10, 15, 16]. Specifically, pregnant women are advised not to consume unpasteurized milk, unpasteurized cheeses, and other foods with *Listeria* species contamination potential [17]. In addition, the excessive use of antimicrobials has led to antibiotic resistance and even multiresistance. The antibiotic resistance of *Listeria* species has undoubtedly made the treatment of listeriosis complex. In the current study area, information on the occurrence, antimicrobial profile, and distribution of *L. monocytogenes* and other *Listeria* species is very limited, although foods of animal origin including milk and milk products are consumed like any other part of Ethiopia. The aims of this study were, therefore, to estimate the prevalence and risk factors of *Listeria* species and determine the antibiogram of *L. monocytogenes* isolated from raw cattle milk and milk products in Ambo, Holeta, and Bako towns, Oromia Regional State, Ethiopia.

## 2. Materials and Methods

### 2.1. Study Area

The study was conducted in three selected towns (Ambo, Holeta, and Bako) of Oromia Regional State, Ethiopia. Ambo town is the administrative center of West Shewa Zone, which is located 114 km West of Addis Ababa at the latitude of 8°59′N 37°51′E and longitude of 8.983°N 37.85°E. The elevation of Ambo town ranges from 1900 to 2275 meters above sea level (m.a.s.l.). The temperature in the town ranges from 19°C to 29°C with an average annual temperature and rainfall of 22°C and 900 mm, respectively. Ambo town has a total human population of 74,843, of which 39,192 are males and 35,651 are female [18]. There were 8 registered dairy farms and many smallholder farms in the town. The total herd size per farm ranged from 3–65 animals. The number of lactating cows per farm ranged from 2 to 32 [19].

Holeta is located in Finfine special zone 44 km West of Addis Ababa with a latitude and longitude of 9°3ʹN 38°30ʹE/9.050ʹN 38.500°E. Its elevation is 2400 m.a.s.l. It receives an annual average precipitation of 1144 mm, with average minimum and maximum temperatures of 6°C and 22°C, respectively. The total human population of Holeta town is 25,593, of which 12,605 are men and 12,988 are women [18]. There are 20 registered farms and many smallholder dairy farms in the town. The herd size per farm ranges from 5 to 300 animals. The lactating cows per farm range from 2 to 140 [20].

Bako town, the capital of Bako Tribe district, is located in the West Shewa Zone of Oromia Regional State. Bako is located 260 km West of Addis Ababa with longitude and latitude of lying between 9.1274°N and 37.0561°E 9°08′N 37°03′E, respectively. It has an elevation ranging from 1300 to 2998 m.a.s.l, an average rainfall of 886.5 mm, and an average temperature of 21.2°C. The town has a total human population of 35,769, of which 16,692 are male and 19,077 are females [18]. There are five registered dairy farms in the town. The total herd size per farm ranges from 7 to 315 animals. The lactating cows per farm range from 2 to 152 [21].

### 2.2. Study Population and Study Design

All lactating dairy cows found in the study area were considered as the study population in this study. A cross-sectional study was conducted from September 2018 to June 2019 in Ambo, Holeta, and Bako towns to estimate the prevalence and associated risk factors of *Listeria* species and the antimicrobial susceptibility profile of *L. monocytogenes* isolated from raw cattle milk and milk products in the study areas.

### 2.3. Sample Size Determination

The sample size was calculated based on the formula given by Thrusfield [22], considering 50% expected prevalence and 95% confidence interval and 5% level of precision. The total sample size calculated using the following formula was 384:

(1) $$\begin{array}{c} N=\frac{1.96^{2}P\;\exp\left(1-P\;\exp\right)}{d^{2}}, \end{array}$$

where *N* = required sample size, *P* = expected prevalence, and *d* = desired absolute precision. This sample size was distributed proportionally to the three towns based on lactating cows' population. Accordingly, 127 lactating cows from Ambo, 190 from Holeta, and 67 from Bako were considered for this study. In addition, a total of 98 samples consisting of bulk tank milk (30), curdled milk “ergo”—naturally fermented curdled milk of a cow (33), and cottage cheese (35) were randomly sampled.

### 2.4. Sample Collection

After identifying dairy farms and milk product selling shops and markets in the study area, a systematic random sampling method was used to collect raw milk (*N* = 384) and milk products (*N* = 98). The raw milk and milk product samples were collected aseptically in sterile plastic test tube (slider freezer bags, oxo-biodegradable, China). Briefly, for milk sample collection from cow, quarters were washed with tap water and soap and dried using individual cow clean towels. After discarding the first three streams of milk, 100 mL of milk was collected aseptically into a sterile test tube that had been prelabeled. After milking all the cows in the farm, 100 mL of bulk tank milk at farm level was collected by blending the milk from all the cows in a milk container together. Samples of bulk tank milk were also collected from collection centers after the milk from different dairy farms were collected and mixed together in the same milk collection tank. In addition, 100 mL curdled milk and about 100 g cottage cheese samples were collected from the sampling locations and kept at 4°C in a sterile universal container.

### 2.5. Study Methodology

#### 2.5.1. Questionnaire Survey

A questionnaire survey consisting of close-ended questions was developed and administered to dairy farm workers of Ambo, Holeta, and Bako towns to assess potential risk factors for milk and milk products contamination by *Listeria* species. From each sample source, one or more members of the household or farm workers who were responsible for milk and dairy product handling were identified and requested to respond to the set of questions. The potential risk factors considered for contamination were age of cows (2–4, 5–7, and 8–15 years), breed (local, cross, or Jersey), parity (first, second, third, or fourth), management (extensive, semi-intensive, or intensive), season (wet or dry), level of education of workers (college/university, secondary, primary, or illiterate), farm hygiene (poor—bad smell and gross filth, moderate—some dirt visible, or good—regularly cleaned and dry), herd size (small ≤10, medium 11–50, or large ≥50), number of lactating cows (≤5, 6–13, or ≥14), farm size (small or large), food safety information (yes or no), training on food safety (yes or no), washing hands before milking (yes or no), washing cow's udder before milking (yes or no), materials used for udder drying (collective towel, individual towel, or just with hand/no towel), source of water for cleaning udder/utensils (pipe or river), washing hands before milking (yes or no), washing udder before milking (yes or no), type of container used to handle milk and milk products (plastic or stainless steel), gender of milk and milk product handler (female or male), and time of udder washing (before milking, no washing, before, or after milking). Bulk milk collectors were also interviewed on how frequently and with what they wash the milk collection tank.

#### 2.5.2. Isolation and Identification of Listeria Species

Raw milk or milk product sample of 25 mL or 25 g, respectively, was measured and agitated thoroughly until the contents became homogeneous. The homogenized samples were then aseptically transferred into flasks containing 225 mL of previously prepared half Fraser (Oxoid, UK) for the sake of repairing Listeria species damaged during transportation. The samples were incubated at 30°C for 24 hours, and then 0.1 mL of the homogenate was taken and inoculated into a 10 mL secondary Fraser broth (Oxoid, UK) and incubated for 24 hours at 37°C. After 24 hours of incubation, 0.1 mL of a positive culture in secondary Fraser broth medium turning a black/dark brown to dark green color were taken and streaked onto Oxford agar (OXA) (Oxoid, UK) plates containing the manufacturer's supplements and the plates were incubated at 37°C for 24–48 hours. After incubation, the growth of Listeria species on the Oxford agar plate was examined for black halo colonies typical of Listeria species. The colonies formed by Listeria species are characterized by a brownish sunken center appearance surrounded by a black halo formation as a result of aesculin hydrolysis. Presumptive Listeria colonies were subcultured on TSAYE (trypticase soy agar with 0.6% yeast extract) (Oxoid, UK), incubated for 24 ± 2 hours at 30°C and further characterized. Typical colonies from TSAYE (1 mm to 2 mm in diameter, convex, colorless, and opaque) were subjected to characterization including Gram staining, motility test, catalase test, hemolysis, carbohydrate utilization, and Christe, Atkins, and Munch–Peterson (CAMP) tests [23, 24]. The Listeria species were assumed to be Gram-positive, Cocco-bacillary or short rod-shaped, catalase positive that forms gas bubbles and umbrella-shaped growth pattern [25].

### 2.6. Antimicrobial Susceptibility Test

Kirby–Bauer disc diffusion method was used for antimicrobial susceptibility testing as per the standard procedure recommended by the Clinical and Laboratory Standards Institute [26]. Antimicrobial susceptibility testing was done for only *L. monocytogenes* due to the limitation of antimicrobial discs. The *L. monocytogenes* isolates were subjected to an antimicrobial susceptibility test against 12 commercially available antimicrobial discs (Oxoid, UK) selected based on common usage. The antimicrobial discs selected were amoxicillin (10 *μ*g), cefotaxime (30 *μ*g), chloramphenicol (30 *μ*g), gentamicin (10 *μ*g), nitrofurantoin (300 *μ*g), azithromycin (15 *μ*g), tetracycline (30 *μ*g), ampicillin (10 *μ*g), vancomycin (10 *μ*g), nalidixic acid (30 *μ*g), oxacillin (1 *μ*g), and norfloxacin (10 *μ*g). Briefly, an inoculum from a cell suspension of approximately 10^6^ cells/mL was used for the test. The cell suspension was prepared by inoculating sterile normal saline with a pure culture of the test organisms and incubating for 4 hours. Following this, the cell suspension turbidity was attuned to an equal 0.5 McFarland Standard. To confirm the matching, the turbidity was also read through a spectrophotometer at 625 nm before inoculation. Two to three colonies of the isolates to be tested in TSAYE were inoculated into the Muller Hinton broth and incubated for 24 hours at 37°C. Then, a swab was taken from each bacterial suspension by sterile cotton swab and then spread on the Muller Hinton agar. According to the standard procedure, the discs were firmly placed in the interval of 3 cm spacing from each other in the medium with sterile forceps and incubated at 37°C for 24 hours [27]. The diameters of clear zones around the discs were measured against a black background using a ruler and compared with standards given by CLSI [26]. *L. monocytogenes* ATCC7644, *Escherichia coli* ATCC25922, and *Staphylococcus aureus* ATCC6538 reference strains were used as quality control.

### 2.7. Data Management and Statistical Analysis

The data generated from laboratory tests and questionnaire surveys were entered into a Microsoft Excel spreadsheet (Microsoft Corporation), transferred, and analyzed using STATA version 14.0 software (StataCorp, College Station, USA). Descriptive statistics was used to summarize the data. The prevalence of *Listeria* species was calculated by dividing the number of positive samples by the total number of tested samples and multiplied by 100. Similarly, the prevalence of antimicrobial-resistant *L. monocytogenes* was calculated by dividing the number of resistant isolates by the total number of tested isolates and multiplied by 100. Chi-square test and logistic regression were used to assess the association of risk factors with the prevalence of *Listeria* species. Dummy variables were created for those explanatory variables with more than two categories. For all risk factors, the level with the lowest prevalence was used as a reference category. Those variables with a *P* value of less than 0.25 in the univariable analysis were further analyzed by multivariable logistic regression after checking for confounders. In all tested cases, 95% confidence intervals and *P* < 0.05 were set for significance.

## 3. Results

### 3.1. Prevalence and Identification of Listeria Species

Out of 482 samples tested, 37 (7.67%; 95% CI: 5.46, 10.42) were found to be positive for the *Listeria* species. The prevalence of *Listeria* species in Ambo town (10.98%) was the highest when compared to Holeta (6.89%) and Bako 3(3.90%) towns (Table 1).

The highest prevalence of *Listeria* species (15.15%; 95% CI: 5.11–31.90) and *L. monocytogenes* (9.09; 95% CI: 1.92–24.33) was detected in bulk tank milk. Similarly, the lowest prevalence of *Listeria* species (6.67%; 95% CI: 0.82–22.07) and *L. monocytogenes* (0.00; 95% CI: 0.00–1.15) was found in curdled milk (Table 2). The overall prevalence of *Listeria* species in milk and milk products was 7.68% (37/482), from which the highest prevalence was recorded for *Listeria grayi* (2.49%) and the lowest for *Listeria weshimeri* (0.62%). The prevalence of *Listeria monocytogenes*, *Listeria ivanovii,* and *Listeria seeligeri* was 2.28%, 1.24%, and 1.04%, respectively.

### 3.2. Risk Factor Analyses

The farm-level prevalence of *Listeria* species was significantly high (*P* < 0.05) in Holeta (63.64%) than Bako (18.75%) towns, in large herd size (85.71%) than smallholder (12.50%), in large farms (58.33%) than small farms (12.50%), and in intensively (50.00%) than extensively (8.33%) managed cows (Table 3). The independent variables, namely, town, management, farm hygiene, education level, herd size, number of lactating cows, farm size, time of udder washing, materials used for udder drying, and source of water for udder and hand washing have a univariable *P* value of less than 0.25; hence, they are considered as potential variables for inclusion into the multivariable model. Among them, the multicollinearity matrix showed that the following variables are collinear: management vs town (*r* = 0.69), education vs management (*r* = 0.51), number of lactating cow's vs farm hygiene (*r* = 0.58), number of lactating cow's vs herd size (*r* = 0.91), farm size vs herd size (*r* = 0.90), and farm size vs number of lactating cows (*r* = 0.83). Among the collinear variables, management, farm hygiene, and herd size were selected for inclusion into the multivariable model due to biological plausibility. Moreover, the time of udder washing, materials used for udder drying, and source of water for udder and utensil washing were also included in the multivariable model (Table 3). Finally, after running the full model, the herd size was removed from the model due to confounding identified through the change in OR >30% between univariable and multivariable models. [28] The Hosmer–Lemeshow goodness-of-fit test revealed that the model predicts or fitted the data well (HLX^2^ = 5.06; *P*=0.7511, sensitivity = 35.3%, specificity = 83.9%, positive predicting value = 54.6%, negative predicting value = 70.3%, ROC = 0.7581). However, none of the variables were independent and significant predictors of *Listeria* species isolation rate.

The univariable logistic regression analysis of animal level risk factors showed that the risk of contamination of raw milk by *Listeria* species was 8.12 times higher in Ambo compared to Bako town (*P*=0.045). Similarly, the likelihood of contamination of milk by *Listeria* species was 2.82 times higher in intensively managed cows compared to extensively managed cows (*P*=0.031). Independent variables like season, breed, age, parity, washing hands before milking, washing udder before milking, materials used for udder drying, and source of water for udder/hand washing were not significantly associated with the isolation of *Listeria* species from raw cow milk (*P* > 0.05). None of the independent variables studied were collinear with each other (*r* < 0.5).

Independent variables with univariable *P* < 0.25 considered for the multivariable model include town, season, breed, management system, materials used for udder drying, and source of water used for udder/hand washing. The final multivariable logistic regression model revealed that the management system is an independent predictor of *Listeria* species isolation from milk (Table 4).

Chi-square analysis of the association between potential risk factors and prevalence of *Listeria* species in bulk tank milk, curdled milk, and cottage cheese revealed that none of the factors considered were significantly associated (*P* > 0.05). In addition, information obtained from the interview made with bulk milk collectors in the study areas identified that containers used for bulk milk collection were washed once using Ajax soap either just before they use it for milk collection or after they finish selling the raw milk from the tank (Table 5).

### 3.3. Antimicrobial Susceptibility

In this study, twelve antimicrobial discs were tested against a total of eleven isolates of *Listeria monocytogenes* for antimicrobial susceptibility test. The isolates showed high resistance to oxacillin (100%), amoxicillin (90.91), and ampicillin (72.73%), whereas the isolates showed 100% susceptibility against gentamycin and norfloxacin. Of the total 11 isolates subjected to antimicrobial susceptibility test, 11 (100%) exhibited resistance for oxacillin, 10 (90.91%) for amoxicillin, and 9 (81.82%) for vancomycin. Moreover, 8 (72.73%) and 7 (63.64%) of the isolates were susceptible to chloramphenicol and nitrofurantoin, respectively (Table 6).

### 3.4. Multidrug Resistance

The majority of the *L. monocytogenes isolates* 9 (81.82%) showed MDR. Out of the 9 MDR *L. monocytogenes* isolates, two isolates showed resistance to 6 classes of drugs (Table 7).

Out of the *L. monocytogenes* isolated from raw milk collected from individual cows (*N* = 7), 5 (71.43%) isolates showed MDR and all isolates from bulk tank milk and cottage cheese showed MDR (Table 8).

## 4. Discussion

*Listeriosis caused by L. monocytogenes* is one of the serious food-borne diseases, especially for people with a weakened immune system. Milk and milk products are the major sources of *L. monocytogenes,* which is of great concern for food quality and safety. In food industry, this pathogen can form biofilms that can resist standard cleaning and disinfection procedures [29]. In the present study, 7.67% and 2.28% of milk and milk products are contaminated with *Listeria* species and *L. monocytogenes,* respectively. The source of contamination of milk and milk products with *Listeria* species and *L. monocytogenes* in the present study may be due to udder infection or contaminated feed and feces [30]. The overall prevalence of *Listeria* species (7.68%) found in the present study is in accordance with the findings of Muhammed et al. [9] who reported a 6.5% prevalence in milk and milk products. In contrast to the present study, the high prevalence of *Listeria* species in raw milk has been reported previously as 25% in Ethiopia [13], 26.6% in Addis Ababa [31], 22.79% in Addis Ababa [14], 26.1% in Addis Ababa [8], 22% in Selale [8], 20.88% in Debre-Birhan [32], and 14% in Jimma [9].

The highest prevalence of *L. monocytogenes* in the present study was found in bulk tank milk (9.09%), followed by cottage cheese (2.85%) and cow-level raw milk (1.82%). *L. monocytogenes* was not isolated from curdling milk perhaps due to the small sample size investigated or better hygienic practice of preparation. In most surveys of raw milk, *L. monocytogenes* prevalence was detected from 1 to 16 % [33–36]. In accordance with the present isolation rate of *L. monocytogenes* (2.28%), Hamdi et al. [37] from Algeria, Aygun, and Pehlivanlar [38] from Turkey and Gebretsadik et al. [8] from Ethiopia reported prevalence of 2.61%, 2.2%, and 4%, respectively. As compared to the present finding, Rahimi et al. [4] from Iran and Ning et al. [39] from China reported a lower prevalence of 1.1% and 0.3–1.2%, respectively. A much higher prevalence of *L. monocytogenes* has also been reported from raw cow milk and milk products from Ethiopia (5.6%) [14] and Botswana (12.3%) [27].

Surveys of *L. monocytogenes* in bulk tank milk from dairy farms in the United States, New Zealand, France, and Belgium showed a prevalence ranging from 2.9 to 6.3 % [33, 40–42], which were lower when compared to the current results from bulk tank milk (9.09%). The present *L. monocytogenes* prevalence from raw milk (1.8%) is lower when compared to the 4% reported from Gonder, Ethiopia [13] but similar to the reports of Seyoum et al. [14] who reported 2.04% prevalence from raw milk of the central highlands of Ethiopia.

The prevalence of *L. monocytogenes* in cottage cheese in the present study (2.85%) was almost similar with the prevalence reported from various parts of Ethiopia ranging from 0 to 5% [8–10, 13, 31, 43, 44]. The heat processing of buttermilk obtained after the churning of sour milk is used to prepare cottage cheese in Ethiopia. The heating practice leads to the precipitation of the protein component of buttermilk and partly might contribute to the low prevalence of *L. monocytogenes* in cottage cheese, which even then, might be due to postprocessing contamination [14]. Most people in the study towns, and elsewhere in the country, eat cottage cheese, trusting that it is heat processed and safe. However, this study demonstrated that this milk product might not be safe unless the public takes precautious measures.

Unlike the previous study by Derra et al. [10] and Gebretsadik et al. [8] who reported a predominant and high isolation rate of *L. innocua* 83%, and 60.8%, respectively, no *L. innocua* was isolated from raw milk and milk products in the present study that was similar to the report in Iran [4]. The variations of *Listeria* species prevalence may be due to differences in risk factors, seasonal variation in milk samples collection, types of samples, or methods of isolation, geographic location, management system, time for conducting the study, level of access to extension services by farmers, and hygienic status of production and processing [13, 14, 44–46].

Analysis of risk factors at the farm level showed that study town, herd size, farm size, the number of lactating cows, and management system were significantly associated with the prevalence of *Listeria* species (*p* < 0.05). However, none of these factors turned out tube predictors of the prevalence of *Listeria* species. The significantly high prevalence of *Listeria* species at farm level in Holeta town (where most large farms and farms with big herd sizes and lactating cows are found) using univariable logistic regression analysis, might suggest the presence of different risk factors contributing to the contamination of milk compared to Bako town.

The high prevalence of *Listeria* species in intensively managed milking cows might be related to the risk of fecal contamination of raw milk by *Listeria* species, which seems to increase during the indoor keeping when the number of fecal excretes, are high since cows are kept together. The effects of the management system on the prevalence *of Listeria* species in housed/indoor cows are higher when compared to outdoor cows, which lead to confinement of the cows and transmission of the organism from infected to healthy ones. In an observational epidemiologic study on the occurrence of *L. monocytogenes* in the feces of dairy cattle, Husu [47] reported that the prevalence of *Listeria* species in raw milk was associated with the prevalence of bacteria in fecal samples. Thus, the fecal excretion of *L. monocytogenes* by cattle was the likely source of contamination of raw milk [48].

The challenge of the pathogenic *L. monocytogenes* is not limited to not only contamination of food items and the environment but also being able to resist the most commonly known antimicrobials that are often used for the treatment of infections. Bacterial resistance to antimicrobial drugs has been rising dangerously to high levels over the world, causing serious public health threats [49]. The use of antimicrobial drugs in low-dose or incomplete courses is the main reason for the emergence and spread of antimicrobial drug resistance. On the other hand, the formation of biofilms on foods, instruments, and utensils and the lack of new antimicrobials being developed can also trigger the ability of the organism to resist the activity of antimicrobials [50]. The problem can be higher in Ethiopia since the consumption of raw milk and raw milk products are very widespread. The relatively high level of antimicrobial-resistant *L. monocytogenes* isolates to oxacillin (100%), amoxicillin (90.91%), ampicillin (72.72%), and cefotaxime (45.45%) are commonly prescribed for the treatment of listeriosis in humans is of great concern. The resistance observed to these drugs might be related to the more frequent prescription, relatively cheaper price, ease of availability, and accessibility by the local community to fight infections in the veterinary and public health sectors [13, 51]. Furthermore, the situation of antimicrobial resistance is further aggravated by the indiscriminate and extensive usage of antimicrobials in the country. The resistance rate to oxacillin in the present study was similar to the reports of Gomez et al. [52] but higher than reports of Ieren et al. [53] (94.1%) and Khen et al. [54] (84.0%). The level of AMR encountered against nalidixic acid (54.55%), tetracycline (36.36%), and chloramphenicol (27.27%) in the current study was comparable to the results of the previous study [13]. Unlike the present findings, Gebremedhin et al. [55] reported a relatively low level of resistance of *L. monocytogenes* to amoxicillin, cefotaxime, vancomycin (5–15%). Previously, Gebremedhin et al. [55] reported that *L. monocytogenes* isolates are resistant to oxacillin (80.0%), nalidixic acid (70%), chloramphenicol (60%), and tetracycline (55%) in the same study area, which is higher than the present findings.

Chloramphenicol, gentamycin, and norfloxacin were the most effective antibiotics since 72.74% to 100% of the isolates were found to be susceptible. This could be because these drugs were the least frequently used in the study areas in veterinary services. Thus, no more resistance was developed. A similar suggestion given by Calderón-Jaimes et al. [56] was the development of antimicrobial resistance is nearly always a result of repeated therapeutic use and/or indiscriminate usage. The use and misuse of antimicrobials in farm animal settings as growth promoters or as means of disease treatment have increased antimicrobial resistance among bacteria in their habitat. This reservoir of resistance may be transferred directly or indirectly to humans through food consumption. The resistant bacteria can cause serious health effects directly or through the transmission of antimicrobial resistance traits to pathogens, causing diseases that are difficult to treat [57].

The high level of resistance (81.82%) to glycopeptide antibiotic (vancomycin) in the present study needs special consideration and serious monitoring due to the possible spread of genes encoding resistance to vancomycin to methicillin-resistant *S. aureus* (MRSA) strains, which are arduous to combat using available therapeutic measures [58]. The presence of a high percentage of MDR *L. monocytogenes* in milk and milk products, which are often consumed raw in Ethiopia, coupled with the inadequate knowledge of people about food-borne infections, inadequate food safety regulatory system [14], the high number of risky groups for listeriosis, and the peculiar features of listeriosis (cold and hot tolerance, opportunistic, and high fatality) suggests that there is a high potential of occurrence and severity of listeriosis in Ethiopia. Thus, integrated hygienic practices from farm to table need to be implemented to tackle the problem.

## 5. Conclusion and Recommendations

The present findings highlight that milk and milk products in the study area are contaminated with *L. monocytogenes* and other *Listeria* species due to inadequate hygienic practices at various levels and potential public health hazards. Study town, herd size, farm size, number of lactating cows, and management system were risk factors for the prevalence of *Listeria* species at farm level, whereas study towns and management system were significant risk factors for isolation of *Listeria* species at cow level. The *L. monocytogenes* showed high degree of resistance to oxacillin, ampicillin, amoxicillin, and vancomycin, whereas the isolates were high susceptibility to gentamycin and norfloxacin. Thus, the latter two drugs could be considered in the clinical management of sick patients. *L. monocytogenes* isolates showed a very high MDR (81.82%). Avoiding the consumption of unpasteurized milk, following hygienic practices to minimize contamination, and rational usage and monitoring of antimicrobial drug usage are recommended, and further studies consisting of serotyping and molecular studies are recommended.

## Figures and Tables

**Table 1:** The overall prevalence of *Listeria* species isolated from dairy, cow milk, and milk products in our study towns.

Study areas	Sample type	*Listeria* species		
		Number tested	Prevalence (%)	95% CI
Ambo	Cow milk	127	14 (11.02)	1.59–4.82
	Curdle milk	10	1 (10.00)	0.00–1.15
	Bulk tank milk	17	3 (17.65)	0.12–1.80
	Cottage cheese	10	0 (0.00)	0.00–1.15
	Subtotal	164	18 (10.98)	2.22–5.83

Holeta	Cow milk	190	12 (6.32)	1.29–4.30
	Curdle milk	15	1(6.67)	0.00–1.15
	Bulk tank milk	7	1 (14.29)	0.00–1.15
	Cottage cheese	20	2 (10.00)	0.00–1.49
	Subtotal	232	16 (6.90)	1.90–5.33

Bako	Cow milk	67	1 (1.49)	0.00–1.15
	Curdle milk	5	0 (0.00)	
	Bulk tank milk	9	1 (11.11)	0.00–1.15
	Cottage cheese	5	1 (20.00)	0.00–1.15
	Subtotal	86	3 (3.49)	0.12–1.80

Overall		482	37 (7.68)	5.46–10.42

CI, confidence interval.

**Table 2:** Comparison of prevalence of *Listeria* species from different samples.

Sample type	Number tested	*Listeria* species		*L. monocytogenes*	
		Number of positive sample	% Prevalence (95% CI)	Number of positive sample	% Prevalence (95% CI)
Bulk tank milk	33	5	15.15 (5.11–31.90)	3	9.09 (1.92–24.33)
Cottage cheese	35	3	8.57 (1.80–23.06)	1	2.85 (0.00–1.15)
Cow level raw milk	384	27	7.03 (4.68–10.07)	7	1.82 (0.58–2.96)
Curdle milk	30	2	6.67 (0.82–22.07)	0	0.00 (0.00–1.15)
Total	482	37	9.64 (6.88–13.04)	11	2.28 (1.14–4.04)

CI, confidence interval.

**Table 3:** Results of logistic regression analysis of farm-level potential risk factors for isolation of *Listeria* species.

Risk factors	Categories	Number tested	Number of positive sample (%)	Univariable		Multivariable	
				Or (95% CI)	*P* value	Or (95% CI)	*P* value
Town	Bako	16	3 (18.75)	1.0	—		
	Ambo	21	7 (33.33)	2.17 (0.46–10.20)	0.328		
	Holeta	11	7 (63.64)	7.58 (1.31–43.92)	0.024		
Farm hygiene	Poor	23	6 (26.09)	1.0		1.0	—
	Good	13	5 (38.46)	1.77 (0.41–7.58)	0.441	1.69 (0.27–10.46)	0.570
	Moderate	12	6 (50.0)	2.83 (0.65–12.26)	0.164	3.53 (0.51–24.59)	0.203
Level of education of workers	College and above	14	4 (28.27)	1.0			
	Secondary	13	4 (30.77)	1.11 (0.21–5.80)	0.901		
	Elementary	15	5 (33.33)	1.25 (0.26–6.07)	0.782		
	Illiterate	6	4 (66.67)	5.0 (0.64–39.06)	0.125		
Herd size	Smallholder (≤10)	24	3 (12.50)	1.0	—		
	Medium (10–50)	17	8 (47.06)	6.22 (1.33–29.01)	0.020		
	Large (≥50)	7	6 (85.71)	42 (3.67–481.03)	0.003		
Farm size	Small	24	3 (12.50)	1.0	—		
	Large	24	14 (58.33)	9.8 (2.28–42.06)	0.002		
Number of lactating cows	≤5	21	2 (9.52)	1.0	—		
	6–13	18	8 (44.44)	7.6 (1.35–42.80)	0.021		
	≥14	9	7 (77.78)	33.25 (3.90–283.45)	0.001		
Management system	Extensive	12	1 (8.33)	1.0		1.0	—
	Semi-intensive	14	5 (35.71)	6.11 (0.60–62.23)	0.126	2.93 (0.18–48.45)	0.452
	Intensive	22	11 (50.00)	11 (1.21–100.39)	0.034	6.38 (0.54–75.95)	0.143
Training on food safety	Yes	23	8 (34.78)	1.0			
	No	25	9 (36.00)	1.05 (0.32–3.45)	0.930		
Washing cows udder before milking	No	7	2 (28.58)	1.0	—		
	Yes	41	15 (36.59)	1.44 (0.25–8.37)	0.683		
Materials used for udder drying	Collective towel	13	2 (15.38)	1.0	—	1.0	—
	Without towel/just with hand	10	4 (40.00)	3.67 (0.51–26.22)	0.196	3.72 (0.26–53.45)	0.333
	Individual towel	25	11 (44.00)	4.32 (0.79–23.68)	0.092	2.52 (0.31–20.52)	0.388
Source of water for udder and utensil washing	River	12	2 (16.67)	1.0	—	1.0	—
	Pipe	36	15 (41.67)	3.57 (0.68–18.72)	0.132	1.53 (0.21–11.11)	0.672
Washing hands before milking	Yes	30	10 (33.33)	1.0	—		
	No	18	7 (38.89)	1.27 (0.38–4.29)	0.697		
Time of udder washing	Before milking	25	7 (28.00)	1.0	—	1.0	—
	No washing	6	2 (33.33)	1.29 (0.19–8.67)	0.796	1.54 (0.16–14.73)	0.710
	Before and after milking	17	8 (47.06)	2.29 (0.63–8.32)	0.210	2.29 (0.46–11.53)	0.314

**Table 4:** Logistic regression analyses of potential risk factors for *Listeria s*pecies isolation rate in study towns at cow level.

Risk factors	Category	Univariable		Multivariable	
		Or (95%CI)	*P* value	Or (95%CI)	*P* value
Town	Bako	1.0	—	1.0	—
	Holeta	4.45 (0.57–34.89)	0.155	3.42 (0.39–29.97	0.267
	Ambo	8.18 (1.05–63.60)	0.045	5.87 (0.71–48.32)	0.100
Breed	Local	1.0	—	1.0	—
	Cross	2.57 (0.58–11.23)	0.209	1.73 (0.33–9.01)	0.513
	Jersey	2.61 (0.42–16.41)	0.305	2.64 (0.39–17.99)	0.323
Age in years	2–4	1.0	—		
	5–7	1.46 (0.55–3.87)	0.448		
	8–15	1.69 (0.52–5.45)	0.382		
Seasons	Wet	1.0	—	1.0	—
	Dry	1.68 (0.72–3.94)	0.232	1.04 (0.30–3.63)	0.951
Management system	Extensive	—	—	1.0	
	Semi-intensive	1.03 (0.34–3.19)	0.943	1.38 (0.37–5.20)	0.635
	Intensive	2.82 (1.10–7.24)	0.031	3.38 (1.02–11.18)	0.046
Parity	Second	1.0	—		
	Fourth	1.09 (0.32–3.69)	0.895		
	First	1.38 (0.49–3.84)	0.544		
	Third	1.65 (0.48–5.67)	0.427		
Washing hands before milking	Yes	1.0	—		
	No	1.37(0.63–3.00)	0.429		
Washing udder before milking	Yes	1.0	—		
	No	1.25 (0.45–3.44)	0.668		
Materials used for udder drying	Collective/shared/towel	1.0	—	1.0	—
	Individual towel	1.36 (0.57–3.23)	0.493	1.47 (0.52–4.11)	
	Just with hand/no towel	2.45 (0.81–7.40)	0.112	2.35 (0.63–8.71)	0.203
Source of water used for udder/utensil washing	River	1.0	—	1.0	—
	Tap	2.09 (0.61–7.14)	0.238	1.70 (0.45–6.41)	0.436

**Table 5:** Results of Chi-square analysis of the association between the prevalence of *Listeria* species in bulk tank milk, curdled milk, and cottage cheese and potential risk factors for contamination.

Variable	Categories	Number tested	Number of positive sample	Prevalence (%)	Chi-square	*P* value
Town	Holeta	42	4	9.52	0.0382	0.981
	Bako	19	2	10.53		
	Ambo	37	4	10.81		
Sample type	Curdle milk	30	2	6.67	1.3931	0.498
	Cottage cheese	35	3	8.57		
	Bulk tank milk	33	5	15.15		
Gender of handler	Female	66	6	9.09	0.2733	0.601
	Male	32	4	12.50		
Level of education	Primary	23	1	4.35	1.2869	0.732
	Illiterate	44	5	11.36		
	Secondary	25	3	12.00		
	College and above	6	1	16.67		
Food safety information	No	32	3	9.38	0.0356	0.850
	Yes	66	7	10.61		
Received training on food safety	No	70	5	7.14	2.5057	0.113
	Yes	28	5	17.86		
Type of container used for handling	Stainless steel	31	3	9.68	0.0137	0.907
	Plastic	67	7	10.45		

**Table 6:** Results of antimicrobial susceptibility testing of *L. monocytogenes* isolates (*N* = 11) from all samples in this study town.

Antimicrobial classes	Antimicrobials	Resistance		Intermediate		Susceptible	
		Number	%	Number	%	Number	%
Glycopeptide	Vancomycin	9	81.82	1	9.09	1	9.09
Aminoglycosides	Gentamycin	0	0	0	0	11	100
Cephem	Cefotaxime	5	45.45	3	27.27	3	27.27
*β*-lactams	Oxacillin	11	100	0	0	0	0
	Ampicillin	8	72.73	0	0	3	27.27
	Amoxicillin	10	90.91	0	0	1	9.09
Quinolones	Nalidixic acid	6	54.55	2	18.18	3	27.27
	Norfloxacin	0	0	0	0	11	100
Nitrofuran	Nitrofurantoin	1	9.09	3	27.27	7	63.64
Macrolide	Azithromycin	3	27.27	4	36.36	4	36.36
Phenicol	Chloramphenicol	3	27.27	0	0	8	72.73
Tetracycline	Tetracycline	4	36.36	0	0	7	63.64

**Table 7:** Multidrug resistance patterns of *L. monocytogenes* isolates (*N* = 11).

Antimicrobial resistance pattern (number of resistant isolates)	MDR pattern	Percent of MDR isolates
OXC-NAL (1)	2	9.1
VAN-OXC-AMP-AMX (1)	2	9.1
VAN-OXC-AMX-TET (1)	3	9.1
VAN-OXC-AZM-CHL-AMX (1)	4	9.1
VAN-CTX-OXC-AMP-AMX (1)	3	9.1
OXC-AMP-NAL-AZM-AMX (1)	3	9.1
VAN-CTX-OXC-AMP-AZM-AMX (1)	4	9.1
VAN-OXC-AMP-NAL-AMX-TET (1)	4	9.1
VAN-CTX-OXC-AMP-NAL-AMX-TET (1)	5	9.1
VAN-CTX-OXC-AMP-NAL-CHL-AMX-TET (1)	6	9.1
VAN-CTX-OXC-AMP-NAL-NIT-CHL-AMX (1)	6	9.1

OXC, oxacillin; NAL, nalidixic acid; VAN, vancomycin; AMP, ampicillin; AMX, amoxicillin; TET, tetracycline, AZM, azithromycin; CHL, chloramphenicol; CTX, cefotaxime, NIT, nitrofurantoin; MDR, multidrug resistance.

**Table 8:** Comparison of antimicrobial resistance profile of *L. monocytogenes* isolated from different sources.

Sample sources	Number of *L.monocytogenes* isolates	Sensitive to all drugs	Intermediate susceptibility	Resistance to single class of drug	Resistance to two classes of drugs	Multidrug resistance (≥3 classes of drugs)
Cow	7	0 (0.00)	4 (57.14)	0 (0.00)	2 (28.57)	5 (71.43)
BTM	3	0 (0.00)	2 (66.67)	0(0.00)	0 (0.00)	3 (100)
CC	1	0 (0.00)	0 (0.00)	0 (0.00)	0 (0.00)	1 (100)
Total	11	0 (0.00)	6 (54.55)	0 (0.00)	2 (18.18)	9 (81.82)

BTM, bulk tank milk; CC, cottage cheese.

## Data Availability

The data sets used and analyzed during the current study are available from the corresponding author upon reasonable request.
